# Machine learning and deep learning algorithms in stroke medicine: a systematic review of hemorrhagic transformation prediction models

**DOI:** 10.1007/s00415-024-12810-6

**Published:** 2024-12-12

**Authors:** Mahbod Issaiy, Diana Zarei, Shahriar Kolahi, David S. Liebeskind

**Affiliations:** 1 Advanced Diagnostic and Interventional Radiology Research Center (ADIR), Tehran University of Medical Sciences, Tehran, Iran; 2 Present Address: Comprehensive Stroke Center and Department of Neurology, David Geffen School of Medicine at the University of California, Los Angeles, Los Angeles, CA USA; 3 Neurovascular Imaging Research Core, University of California, Los Angeles, Los Angeles, CA USA

**Keywords:** Acute ischemic stroke, Hemorrhagic transformation, Machine learning, ML, DL, Systematic review

## Abstract

**Background:**

Acute ischemic stroke (AIS) is a major cause of morbidity and mortality, with hemorrhagic transformation (HT) further worsening outcomes. Traditional scoring systems have limited predictive accuracy for HT in AIS. Recent research has explored machine learning (ML) and deep learning (DL) algorithms for stroke management. This study evaluates and compares the effectiveness of ML and DL algorithms in predicting HT post-AIS, benchmarking them against conventional models.

**Methods:**

A systematic search was conducted across PubMed, Embase, Web of Science, Scopus, and IEEE, initially yielding 1421 studies. After screening, 24 studies met the inclusion criteria. The Prediction Model Risk of Bias Assessment Tool (PROBAST) was used to assess the quality of these studies, and a qualitative synthesis was performed due to heterogeneity in the study design.

**Results:**

The included studies featured diverse ML and DL algorithms, with Logistic Regression (LR), Support Vector Machine (SVM), and Random Forest (RF) being the most common. Gradient boosting (GB) showed superior performance. Median Area Under the Curve (AUC) values were 0.91 for GB, 0.83 for RF, 0.77 for LR, and 0.76 for SVM. Neural networks had a median AUC of 0.81 and convolutional neural networks (CNNs) had a median AUC of 0.91. ML techniques outperformed conventional models, particularly those integrating clinical and imaging data.

**Conclusions:**

ML and DL models significantly surpass traditional scoring systems in predicting HT. These advanced models enhance clinical decision-making and improve patient outcomes. Future research should address data expansion, imaging protocol standardization, and model transparency to enhance stroke outcomes further.

**Supplementary Information:**

The online version contains supplementary material available at 10.1007/s00415-024-12810-6.

## Introduction

Stroke, particularly acute ischemic stroke (AIS), continues to be a major contributor to morbidity and mortality globally, placing a substantial burden on healthcare systems [1]. A critical complication following AIS is hemorrhagic transformation (HT), wherein ischemic brain tissue undergoes secondary bleeding. This process exacerbates neurological deficits and elevates the risk of mortality [2]. This complication typically occurs following the reperfusion of cerebral tissue, often due to thrombolytic therapies [3]. Therefore, accurate prediction of HT is crucial for optimizing therapeutic strategies and enhancing patient outcomes.

The integration of artificial intelligence (AI) into medical research holds the potential to revolutionize the prediction and management of stroke outcomes. Over the years, AI has evolved significantly, transitioning from early rule-based systems to advanced machine learning (ML) and deep learning (DL) algorithms [4]. AI encompasses the development of algorithms and computational models that mimic human cognitive functions. Within AI, ML, and DL have emerged as powerful tools in medical research, offering the capability to analyze vast amounts of data and identify patterns that traditional statistical methods may overlook [5]. Predictive models in stroke medicine are designed to estimate the risk of complications such as HT based on various patient-specific factors. Traditional models have relied on clinical, radiological, and laboratory data; however, their predictive accuracy is often limited by the complexity and heterogeneity of stroke presentations. This limitation underscores the need for more sophisticated approaches capable of handling multifaceted data and extracting meaningful patterns. Examples of such scoring systems include the Hemorrhage After Thrombolysis (HAT) score, the Safe Implementation of Treatments in Stroke Symptomatic Intracerebral Hemorrhage (SITS-SICH) risk score, and the Stroke Prognostication using Age and National Institutes of Health Stroke Scale-100 index (SPAN-100) [6].

ML, a subset of AI, focuses on developing algorithms that can learn from data and make predictions or decisions without being explicitly programmed. ML techniques, such as support vector machines (SVM), random forests (RF), and logistic regression, have been extensively applied in medical research to predict clinical outcomes. These algorithms excel in supervised learning scenarios where labeled data is available for training [5].

DL, which falls under the broader category of ML, utilizes a layered approach to analyze and learn from data [7]. This enables it to identify intricate patterns and relationships within datasets. Convolutional neural networks (CNNs) and recurrent neural networks (RNNs) are prominent DL architectures. CNN has proven to be especially effective in the analysis of medical images and shows great potential in making clinical predictions based on imaging data [8]. RNNs are adept at handling sequential data, making them suitable for time-series prediction tasks such as monitoring patient vital signs [9].

Recent studies have shown a marked increase in the application of these algorithms in areas such as stroke research [10, 11]. ML has been particularly successful in predicting HT, as evidenced by various studies [12].

Considering the life-threatening consequences of HT after AIS, and the emergence of numerous ML and DL tools designed to predict the risk of HT based on different inputs this study aims to systematically review and evaluate these predictive models. Our review will focus on comparing the efficacy of these algorithms and, where feasible, benchmark them against existing scoring systems. This comparison will not only highlight the potential of ML and DL to enhance predictive accuracy but also identify areas where these technologies could be refined for better integration into clinical workflows. Ultimately, our review endeavors to underscore the significance of leveraging cutting-edge technologies to improve patient outcomes in AIS, setting the stage for future innovations in stroke management.

## Methods

### Study design

This study is a systematic review to evaluate the accuracy and efficacy of ML and DL algorithms in predicting HT following AIS. The review follows the Preferred Reporting Items for Systematic Reviews and Meta-Analyses (PRISMA) guidelines, detailed in Table S1 [13]. Furthermore, this study's protocol has been registered with the International Prospective Register of Systematic Reviews and assigned the identification number CRD42023492308.

### Search strategy

In this systematic review, we executed an extensive and detailed search across multiple databases, including PubMed, Embase, Scopus, Web of Science (ISI), and IEEE. This search was strategically focused on keywords associated with “ischemic stroke”, “machine learning”, and “hemorrhagic transformation”, to identify pertinent literature up to May 18, 2024. For a detailed insight into the search techniques used for each database, please consult the comprehensive explanation available in Table S2.

### Study selection and eligibility criteria

Initially, two reviewers independently screened the search results, examining titles and abstracts. They then conducted a detailed review of the full-text articles to determine their relevance. In instances of disagreement, a third reviewer was consulted to provide an additional opinion. Inclusion criteria were original, peer-reviewed research articles in English that developed and validated ML and/or DL models for HT risk prediction after AIS. Exclusion criteria included studies using external databases, lacking detailed methodologies, unavailable in full text, or not focusing on HT prediction. Literature types such as case reports, reviews, conference proceedings, and editorials were also excluded.

### Data extraction

Two reviewers independently extracted data into a Google Sheet, consulting a third reviewer in case of disagreements. The extracted information covered various aspects, including study design, patient details, data sources, eligibility criteria, sample demographics, treatment types, definitions and assessment timings of HT, features for model training, types of algorithms, preprocessing methods, model structure, comparison models, scoring systems, and the area under the curve (AUC) as the key performance indicator, alongside principal findings, limitations, and suggestions for future research.

### Risk of bias assessment

The studies were assessed using the Prediction Model Risk of Bias Assessment Tool (PROBAST), designed for bias risk evaluation in four domains and the suitability of diagnostic and prognostic models [14]. This assessment was independently conducted by two authors, with a third resolving any differences. Using PROBAST criteria, studies were categorized into low, unclear, or high bias risk, with a high-risk designation applied if significant bias was identified in any of the four domains.

### Data synthesis and analysis

In this systematic review, we conducted a qualitative synthesis to compare and contrast the outcomes of various ML and DL models, alongside traditional scoring systems. Our study's broad scope, encompassing diverse ML and DL models and input variables, renders a meta-analysis impractical due to the required homogeneity in methods and metrics. Instead, our focus is on a comparative evaluation, examining the performance, adaptability, and practicality of these computational models.

## Results

**Fig. 1:**
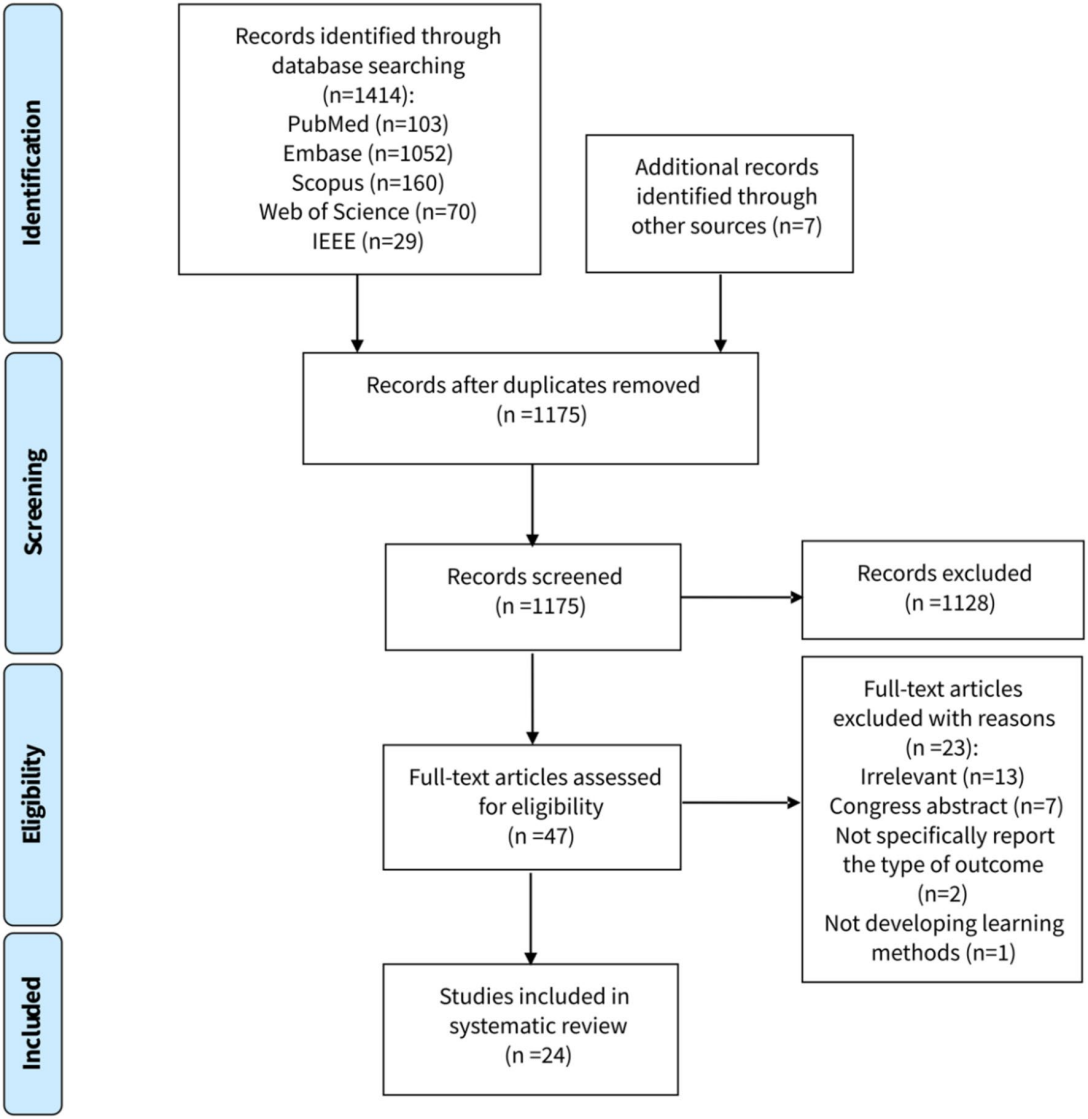
Study selection

We identified 1414 studies by searching designated databases and found seven more studies through cross-referencing. Removing duplicates resulted in 1175 unique studies. Initial screening narrowed these down to 47 articles, and after a detailed review, 24 were chosen for inclusion. The selection process is depicted in Fig. 1, showcasing the PRISMA flowchart.

### Risk of bias assessment

Two independent reviewers rigorously evaluated the integrity of the included studies, resolving any discrepancies with the help of a third reviewer using the PROBAST tool. Among 24 studies examined, seven were found to have a high risk of bias, mainly due to issues in participant selection and analysis methods [15–21]. The comprehensive quality assessment results are delineated in Table S3.

### Study characteristics

Among the included studies, the range of publication years extends from 2012 [18] to 2024 [22, 23]. Most of the studies, specifically 21 out of 24 (87%), were conducted starting from 2020. Methodologically, 21 (87%) studies adopted a retrospective design [10, 15–34], and only three were conducted with a prospective design [12, 35, 36]. The geographical distribution of the included studies predominantly featured research conducted in China, with 14 out of 24 studies (58%) originating from this region [10, 15–17, 21, 22, 24, 26–30, 33, 34], followed by South Korea, contributing four studies [20, 23, 25, 31]. Additional contributions came from various countries, each represented by a single study: The United Kingdom [19], Germany [12], Italy [36], Egypt [35], Taiwan [32], and Thailand [18].

### Demographics

The sample sizes across the studies varied considerably, with the smallest cohort comprising 43 individuals [36] and the largest encompassing 146,062 participants [12]. The median sample size across these investigations was 350, with an interquartile range (IQR) from 129 to 1118, illustrating a significant disparity in study scales. Most studies, 19 out of 24 (79%), reported sample sizes below 2000 [10, 15–26, 28, 30, 32, 34–36].

Regarding demographic details, all studies except for two [18, 23] provided the mean age of the participant populations. The mean age spanned from 62.8 years [35] to 77 years [36], with a median age across studies being 69.22 years and an IQR from 66.6 to 71.3 years. The age distribution revealed that the majority of studies (18 studies, 82%) reported mean ages within the 65 to 75 years’ bracket. Only one study [36] reported a mean age over 75 years, while three studies [17, 26, 35] reported mean ages below 65 years.

The gender distribution across studies that reported the gender of included participants (22 studies) indicated a male predominance, with male-to-female ratios extending from 1.03 to 2.55, and a median value of 1.62 (IQR 1.28–1.95)—most of the studies, 17 out of 22 (77%), documented male-to-female ratios less than 2 [12, 15, 16, 19, 20, 22–25, 28–33, 35, 36].

Regarding treatment modalities, out of the 24 studies analyzed, 19 detailed the type of treatment administered, which included intravenous thrombolysis (IVT), endovascular thrombectomy (EVT), or both. IVT emerged as the most frequently utilized intervention in 12 studies [16, 18, 19, 21, 24, 26–30, 32, 33], followed by EVT in four studies [15, 25, 34, 36], EVT + IVT in two studies [12, 20], and EVT or IVT in one study [23]. One study investigated the risk of HT in patients who arrived late and did not undergo any treatment [22].

Further details regarding the characteristics of the included studies are summarized in Table 1.

**Table 1:** Detailed characteristics of included studies on machine learning algorithms for predicting hemorrhagic transformation following acute ischemic stroke

Study, country (year), study design	Interval time for evaluation	Treatment type	Sample size	Age (years), mean ± SD/median (IQR)	Sex (M/F)	# HT
Heo et al., South Korea (2024) [23], Retro	w/i 24 h	EVT or IVT	362	Median: 77 (IQR 69–83)	185/177	218
Huang et al., China (2024) [22], Retro	w/i 7 days	No treatment	140- > train: 99, val.: 41	65.2 ± 12.2	86/54	59
Wen et al., China (2023) [34], Retro	w/i 72 h	EVT	105- > train: 73, int. val.: 32	Train: 72.0 ± 13.1 Int.val: 71.4 ± 13.9	66/29	52
Ren et al., China (2023) [24], Retro	w/i 36 h	IVT	517- > train: 355, int. val.: 90, ext. val.: 72	67.02 ± 12.67- > train: 67.19 ± 12.39, int. val.: 66.83 ± 12.15, ext. val.: 66.44 ± 14.70	333/184	249
Ru et al., China (2023) [16], Retro	w/i 12–36 h	IVT	828	Non-HT: 67 (59–77), HT: 70 (62–82)	547/281	69
Heo et al., South Korea (2023) [25], Retro	w/i 72 h	EVT	202	71.4 ± 14.5	103/99	109
Li et al., China (2023) [26], Retro	NR	IVT	Cohort 1: 1182 (train and int. val.), cohort 2: 227 (ext. val.)	Cohort 1: 62.82 ± 11.53 Cohort 2: 62.90 ± 11.43	Cohort 1: 835/347 Cohort 2: 165/62	Cohort 1: 587 Cohort 2: 112
Ros et al., Italy (2023) [36], Pros	w/i 24 h	EVT	43	77 (69–83)	26/17	23
Jiang et al., China (2023) [15], Retro	w/i 24 h	EVT	Dataset 1 (338)- > train (75%): HT: 187, non-HT: 66, test (25%): HT: 63, non-HT: 22, Dataset 2 (54) for ext. val	Dataset 1, HT: 70.9 ± 11.2, Dataset 1, non-HT: 65.7 ± 9.6, Dataset 2, HT: 71.3 ± 10.1, Dataset 2, non-HT: 64.2 ± 8.5	Dataset 1: 213/125 Dataset 2: 31/23	Dataset 1: 88 Dataset 2: 15
Wen et al., China (2023) [27], Retro	w/i 36 h	IVT	Train (80%) and int. val. (20%): 6369, ext. val.: 1921	Train and int. val.: 65 (57, 71), ext. val.: 65 (58, 72)	5858/2429	121
Bonkhoff et al., Germany (2022) [12], Pros	NR	IVT: 24989 / Intra-arterial thrombectomy + thrombolysis: 10706	146062 (dev. cohort: 74749, Val. cohort: 71,313)	72.7 ± 13.1	76,828/69234	2580
Elsaid et al., Egypt (2022) [35], Pros	w/i 7 days	NR	354- > train: 177, test: 177	62.8 ± 10.5	199/155	70
Xu et al., China (2022) [28], Retro	w/i 48 h	IVT	345- > Train: 80%,val.: 20%	70 (63–81)	224/121	45
Liu et al., China (2022) [29], Retro	w/i 36 h	IVT	1738 Caucasians for training and 296 Han Chinese pts to validate	Caucasian: 68.37 ± 12.62, Chinese: 69.39 ± 13.37	Caucasian: 1016/622, Chinese: 165/131	114
Meng et al., China (2022) [17], Retro	w/i 72 h	NR	71- > train: 49, val: 22	Non-HT: 64 (range 40–85), HT: 64 (range 41–83)	51/20	11
Cui et al., China (2022) [30], Retro	w/i 36 h	IVT	517- > train: 332, int. val.: 83, ext. val.: 102	67.02 ± 12.67	333/184	NR
Xie et al., South Korea (2022) [20], Retro	w/i 7 days	IVT, EVT, IVT + EVT	118- > train: 83, test: 35	69.22 ± 12.33	63/55	52
Wang et al., China (2022) [21], Retro	NR	IVT	144 (288 NCCT)	70.06 ± 11.3	NR	88
Choi et al., South Korea (2021) [31], Retro	w/i 48 h	NR	2028- > train: 1419, test: 609	Total- > 69.6 ± 12.8, train: 69.7 ± 12.9, test: 69.3 ± 12.4	1183/845	318
Chung et al., Taiwan (2020) [32], Retro	w/i 72 h	IVT	331	69.2 ± 12.2	198/133	25
Wang et al., China (2020) [33], Retro	w/i 24 h	IVT	2237- > train and int. val.: 70:30%	Non-HT: 66.32 ± 12.67, HT: 69.54 ± 11.89	1438/799	102
Yu et al., China (2018) [10], Retro	w/i 24 h	NR	62	HT group: 71 ± 13, non-HT group: 67 ± 13	42/20	41
Bentley et al., UK (2014) [19], Retro	w/i one week	IVT	116- > train: 106, val.: 10	HT: 75.1 (95% CI: 69.3–80.9), non-HT: 73.2 (95% CI: 70.7–75.7)	59/57	16
Dharmasaroja et al., Thailand (2012) [18], Retro	W/i 36 h	IVT	194	NR	NR	NR

*EVT* endovascular treatment, *F* female, *h* hour, *HT* hemorrhagic transformation, *IVT* intravenous thrombolysis, *IQR* interquartile range, *M* male, *NR* not reported, *Pros* prospective, *Retro* retrospective, *SD* standard deviation

### Algorithms

The analysis revealed LR as the most frequently used learning algorithm, applied in 14 studies [10, 12, 20, 22–24, 26–28, 30, 31, 33–35], followed by SVM in 10 studies [10, 18, 19, 24, 27, 29–31, 33, 35], and RF in eight studies [17, 24, 26–28, 30, 33, 35]. Yu et al. utilized the broadest array of ML algorithms, encompassing six distinct models [10]. In contrast, the research conducted by Wang et al. [33], Elsaid et al. [35], Wen et al. [27], and Ren et al. [24] involved the application of five distinct algorithms. Another four studies [15, 16, 21, 25] utilized some form of CNN, either as the main component or in some part of their pipeline.

Among the studies, all conducted internal validation to assess their models' performance, but only nine also carried out external validation [15, 20, 24–27, 29, 30, 33].

### Input variables

Clinical features were used as input variables in 18 studies, and imaging findings were employed in 16 studies. Twelve studies [16, 18–25, 30, 34, 36] employed computed tomography (CT) images, either as a direct input [16, 19–21, 25, 30, 36] or using extracted features [18, 22–24, 34] (e.g., radiomics). Four studies incorporated features derived from magnetic resonance imaging (MRI) in their analyses [10, 15, 17, 35]. Jiang et al. [15], Meng et al. [17], and Yu et al. [10] utilized multiparametric MRI, integrating various imaging sequences to enhance predictive capabilities. Conversely, Elsaid et al. [35] applied a comprehensive suite of conventional MRI sequences (e.g., T1, T2, FLAIR). Of the aforementioned studies, two [17, 35] utilized the extracted features from these imaging techniques, while the remaining two [10, 15] employed these imaging modalities as direct inputs.

### Algorithm-level performance

We report the performance of different algorithms using AUCs. LR (without regularization) was reported 10 times with a median AUC of 0.77 [IQR 0.71, 0.81], while LR with LASSO regularization was reported twice with AUCs of 0.80 and 0.87. SVM was used 10 times across studies with a median AUC of 0.767 [IQR 0.73, 0.87]. Similarly, RF was utilized 10 times with a median AUC of 0.831 [IQR 0.76, 0.91]. Next in line, gradient boosting (GB) was used seven times across multiple studies with a median AUC of 0.91 [0.8, 0.94]. Neural networks (artificial neural network (ANN), multilayer perceptron (MLP), and probabilistic neural network (PNN)) appeared eight times throughout the studies with a median AUC of 0.81 [0.78, 0.84]. Cumulatively, CNNs were used 21 times, albeit most of them were reported in a single study [15], with a median AUC of 0.91 [IQR 0.82, 0.93].

### Algorithmic performance compared to traditional scoring systems

**Fig. 2:**
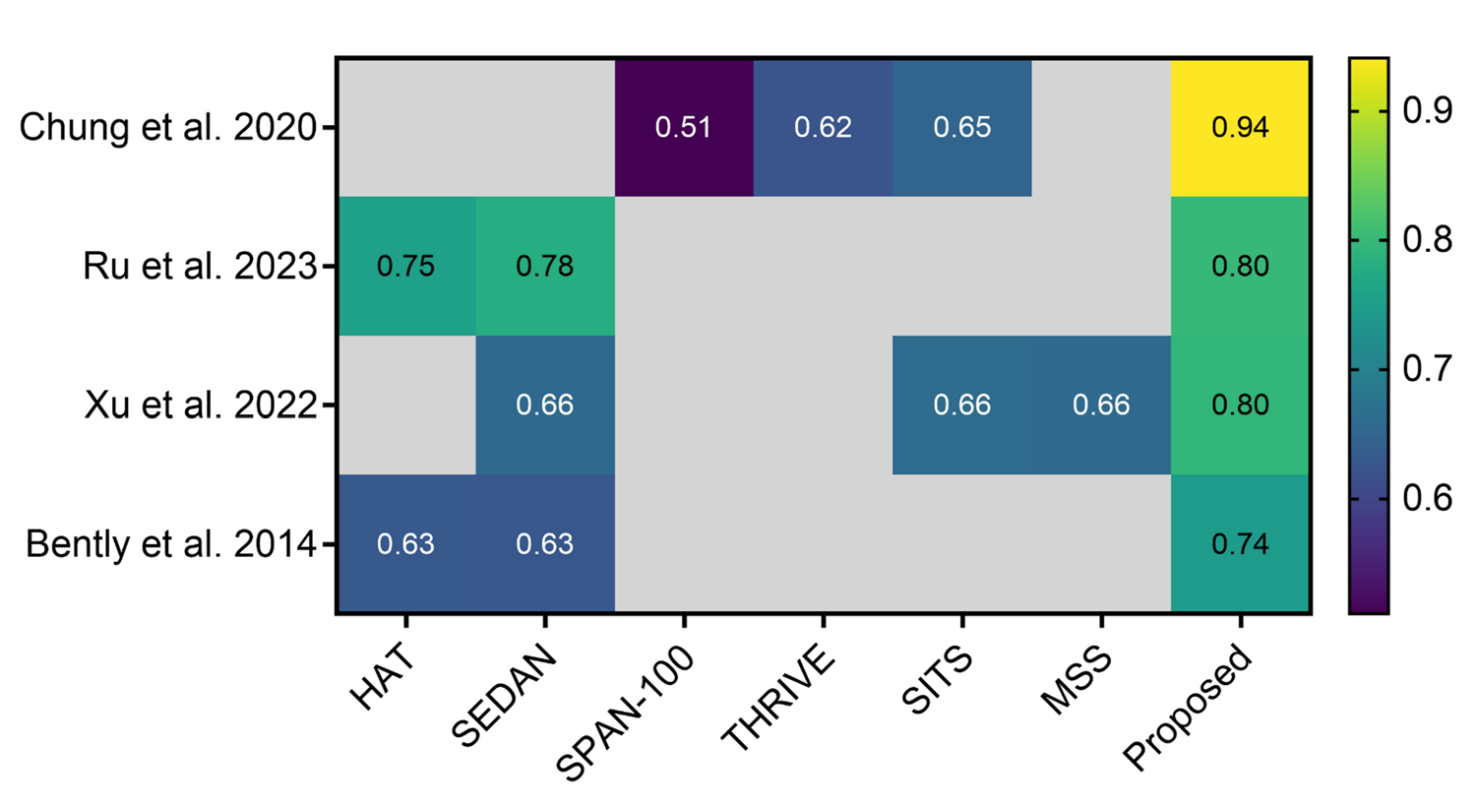
Assessment of proposed algorithm performance versus traditional scoring systems in predicting hemorrhagic transformation following acute ischemic stroke

Four studies compared their proposed algorithms to traditional scoring systems [16, 19, 28, 32]. The scoring systems included HAT, SEDAN, SPAN-100, THRIVE, MSS, and SITS. All of them showed that their proposed ML model outperformed the scoring systems. Particularly notable was the study by Chung et al. [32], where their method achieved an AUC of 0.94 compared to the best-performing scoring system, SITS, which scored 0.65. Figure 2 illustrates a graphical depiction of these comparisons.

Ru et al. [16] incorporated both non-contrast CT (NCCT) and clinical information, Chung et al. [32] and Xu et al. [28] developed their models only on clinical information, and Bentley et al. [19] solely relied on NCCT as input.

### Study-level performance

Across the analyzed studies, ensemble and advanced ML techniques, particularly RF and GB, exhibited outstanding performance. In a recent study by Heo et al., the GB model utilizing radiomics features achieved an impressive AUC of 0.98 [23]. Noteworthy results were also reported by Ren et al. [24] who achieved an AUC of 0.94 with GB using a combination of clinical and radiomics features, while Li et al. [26] demonstrated an AUC of 0.95 with the same algorithm but applied to different laboratory findings. Furthermore, Cui et al. [30] identified Extreme GB (XGB) as the top-performing model, achieving an AUC of 0.91.

DL approaches, particularly those incorporating CNNs and attention mechanisms, showed promising results. Ru et al. [16] developed weakly-supervised DL (WSDL), a model integrating a pre-trained CNN with attention-based pooling, achieving an AUC of 0.8. Heo et al. [25] utilized a 3D CNN, obtaining an AUC of 0.91. Jiang et al. [15] and Wang et al. [21] explored novel neural network architectures, though with varying degrees of success, highlighting the potential and challenges of DL methods in this domain.

Studies utilizing traditional ML algorithms revealed a broad spectrum of outcomes. For instance, Wen et al. [27] and Bonkhoff et al. [12] highlighted the effectiveness of LR with L1-regularization, achieving AUCs of 0.87 and 0.80, respectively. Conversely, Xu et al. [28] found a non-significant difference between RF and LR performances. Notably, Meng et al. [17] and Elsaid et al. [35] reported high AUCs (0.91) using RF and GB models.

Ren et al. [24] and Meng et al. [17] consistently found that integrating clinical and imaging data as inputs for ML models led to improved performance.

Liu et al. [29] presented an interesting finding with their SVM model, showing a considerable disparity in performance between Caucasian and Chinese patient populations (AUC of 0.87 vs. 0.74, respectively).

Table 2 comprehensively presents the specific algorithms utilized within each study, detailing their respective inputs and various attributes.

**Table 2:** In-depth overview of machine learning methodologies implemented in the included studies and their performance

Study (year)	Algorithm	Input variables	Preprocessing methods	Missing data handling/imbalance addressing/regularization technique	Model performance, AUC (95% CI)
Heo et al. [23] (2024)	LightGBM (with all features), Extratrees (with textural features), LR (clinical variables)	Clinical variables and radiomics features from NCCT	Included data conversion, VOI definition, normalization, windowing, resampling, and feature extraction	KNN for clinical data/NR/10fold cross-validation	LightGBM (test set) → 0.986 (0.971–1.00) Extratrees → (test set): 0.845 (0.774–0.916) LR → 0.544 (0.431–0.658)
Huang et al. [22] (2024)	LR	Clinical variables and radiomics features from NCCT	Normalization, segmentation, resampling, feature extraction, feature selection	NR/ Apply instance-level data augmentation techniques: flipping, scaling, rotation, cropping/3fold cross-validation	Clinicoradiomics nomogram model: train → 0.86 (0.78–0.93), val. → 0.90 (0.80–1.00) Clinical model: train → 0.64 (0.56–0.72), val. → 0.63 (0.50–0.76)
Wen et al. [34] (2023)	MLRA	Clinical variables and radiomics features from NCCT	Intensity normalization, imaging interpolation, gray level discretization, manual segmentation: MCA territory regions of interest	NR/ Anonymize patient data in DICOM headers/ NR	Train → 0.781 (0.675–0.886) int.val. → 0.797 (0.642–0.951)
Ren et al. [24] (2023)	SGD, SVM, LR, RF, XGB	Clinical variables and radiomics features from NCCT	Normalization, resampling, standardization	Standardize DECT images: Resize to 256 × 256 × 36 with center crop or edge padding. Convert HU to voxel values; normalize using the external dataset's mean and SD/5fold cross-validation	Training cohort → SGD 0.912, SVM: 0.936, LR: 0.874, RF: 0.926, XGB: 0.953, XGB in training cohort (clinical features only): 0.996 (0.991–0.999), (radiomics only): 0.999 (0.999–1.000), Combined: 0.995 (0.991–0.999), XGB in int. val. cohort → (clinical features only): 0.898 (0.873–0.921), (radiomics only): 0.922 (0.896–0.941), (combined): 0.950 (0.925–0.967), XGB in ext. cohort → (clinical only): 0.911 (0.891–0.928), (radiomics): 0.883 (0.851–0.902), (combined): 0.942 (0.927–0.958)
Ru et al. [16] (2023)	WSDL	NCCT images and clinical variables	Image Preprocessing: Piecewise sampling, 256 × 256 resampling, window adjustments, channel augmentation. Clinical data: normalize, and use ImageNet pre-trained DCNN	Exclusion of tests with high missing rates and outliers. Remaining missing data handled using median, mean, or mode, depending on data characteristics. Normalization of certain data elements for consistency/NR/NR	WSDL: 0.799 (0.712–0.883)
Heo et al. [25] (2023)	3D CNN (Utilized a 3D CNN w/3D ResNet structure)	DECT	NR	Multiple Imputation by Chained Equations (MICE)/SMOTE/ Backward stepwise regression for LR	Train: 0.867 (0.827–0.867), Test: 0.911 (0.774–1.000)
Li et al. [26] (2023)	XGB, LR, RF, DT	Laboratory results	NR	NR/ Down-sampling addresses imbalance, creating samples with 50% affected and 50% non-affected patients/NR	Int. val. → XGB: 0.95 (0.93–0.96), DT: 0.90 (0.88–0.91), RF: 0.91 (0.89–0.92), LR: 0.82 (0.80–0.85)
Da Ros et al. [36] (2022)	Bernoulli Naive Bayes Classifier	CB-CT	Offset correction, gain correction, scatter correction, and water beam-hardening correction	NR/NR/NR	0.876
Jiang et al. [15] (2023)	CNN models w/ both single-parameter and multi-parameter approaches	Multi-parametric MRI data (DWI, MTT, TTP, CBF, CBV) and clinical data	Images processed w/ Pixel intensity normalization Linear compression to [0, 255] range Histogram Equalization Saved in PNG format Clinical data processing: Normalized using min–max normalization	NR/NR/10fold cross-validation	VOI dataset = > single parameter- > clinical: 0.680, DWI: 0.830, MTT: 0.933, TTP: 0.916, CBF: 0.835, CBV: 0.878 multi-parameters model- > MT: 0.924, MTC: 0.924, DMT: 0.933, DMTC: 0.948, DMTC*: 0.939, Slice dataset = > single parameter- > clinical: 0.680, DWI: 0.609, MTT: 0.945, TTP: 0.889, CBF: 0.689, CBV: 0.702 multi-parameters model- > MT: 0.896, MTC: 0.913, DMT: 0.921, DMTC: 0.932, DMTC*: 0.927
Wen et al. [27] (2023)	LR w/o regularization (reference model), LR w/ LASSO regularization, SVM, RF, GBDT, MLP	Clinical variables and therapeutic metrics	NR	5-nearest neighbor model/SMOTE/5fold cross-validation	Ext. val. → Reference model: 0.575 (0.44–0.71), LR w/ LASSO: 0.87 (0.79–0.95), SVM: 0.582 (0.472–0.692),RF: 0.536 (0.42–0.653), GBDT: 0.436 (0.305–0.568), MLP: 0.766 (0.637–0.894)
Bonkhoff et al. [12] (2022)	LR, L1-regularized LR, KNN, GB	Clinical variables	Down-sampling step	NR/SMOTE/10fold cross-validation	Val. → LR: 0.79 (0.79– 0.79), L1-regularized LR: 0.80 (0.79– 0.80), KNN: 0.78 (0.78– 0.78), GB: 0.80 (0.80– 0.80)
Elsaid et al. [35] (2022)	LR, SVM, RF, GB, MLP	Clinical variables, laboratory findings, and MRI markers	NR	MissForest algorithm/NR/NR	Test → GB: 0.91 (0.86–0.95), RF: 0.91 (0.87–0.96), SVM: 0.90 (0.85–0.94), LR: 0.84 (0.77–0.91), MLP: 0.85 (0.78–0.92)
Xu et al. [28] (2022)	RF, LR	Clinical variables and laboratory results	NR	NR/NR/NR	Val. → RF: 0.795 (0.647–0.944), LR: 0.703 (0.515–0.892)
Liu et al. [29] (2022)	SVM	Clinical variables and laboratory results	Selecting the top-8 predictive features using RF	NR/NR/NR	Caucasian cohort: 0.87 (0.83–0.91), Chinese cohort: 0.74 (0.64–0.83)
Meng et al. [17] (2022)	RF	Radiomics features extracted from MRI and clinical variables	NR	NR/NR/5fold cross-validation	Clinical model: 0.556 ± 0.045, Radiomics model w/ abnormal ROI: 0.831 ± 0.006, radiomics model w/ all ROIs: 0.831 ± 0.006, combined model: 0.911 ± 0.009
Cui et al. [30] (2022)	LR, RF, SVM, XGB	Clinical variables, laboratory results, and CT findings	Normalize data: Zero mean, unit variance Feature selection: Lasso post-univariate analysis	NR/NR/Data augmentation (rotation, shifts, zoom), mini-batch size setting for preventing overfitting, and 5fold cross validation	XGB: 0.914, LR: 0.908, RF: 0.894, SVM: 0.893
Xie et al. [20] (2022)	LR	NCCT	Image normalization, lesion segmentation, ROI normalization, resampling, smoothing, and fixing bin width	NR/ Implement dynamic oversampling for the HT dataset/NR	0.750 (0.585–0.915)
Wang et al. [21] (2022)	DBSE-Net	NCCT	NR	NR/random oversampling/10fold cross-validation	0.720
Choi et al. [31] (2021)	LR, SVM, XGB, ANN	Clinical variables	One-hot encoding applied. Scaling techniques used: normalization, min–max scaling, standardization, robust scaling	Use Missing-Indicator Technique for variables with > 5% missing data/ Over-sampling method and cost-sensitive adaptation/NR	ANN: 0.84, SVM: 0.73, BLR: 0.75, XGB: 0.74
Chung et al. [32] (2020)	ANN	Clinical variables	NR	NR/random sampling/10fold cross-validation	Train: 0.951 ± 0.02, Val.: 0.941 ± 0.03
Wang et al. [33] (2020)	RF, LR, NN, SVM, AdaBoost	Clinical variables	Imputation of missing values, normalization, and imbalance processing	NR/ensured equal counts of bleeding and non-bleeding samples/10fold cross-validation	NN: 0.82, SVM: 0.79, LR: 0.77, AdaBoost: 0.77, RF: 0.76
Yu et al. [10] (2018)	SR-KDA, SR-DA, SVM, LR, DT, feedforward NN	Pre-intervention contrast time-curve from PWI, AIF, and DWI values	Co-registration of images w/ SPM12 Automatic AIF detection via Olea Sphere Bilinear interpolation of AIF/PWI Median filter application for noise reduction	NR/down-sampling non-sICH group/NR	SR-KDA: 0.837 ± 0.026, SVM: 0.821 ± 0.029, NN: 0.807 ± 0.043, DT: 0.798 ± 0.031, SR-DA: 0.751 ± 0.036, LR: 0.585 ± 0.075
Bentley et al. [19] (2014)	SVM	NCCT	Global mean intensity adjusted in images Excluded or replaced anomalous voxels w/ abnormal values	NR/NR/10fold cross-validation	SVM: 0.744 (0.738–0.748)
Dharmasaroja et al. [18] (2012)	RBF, MLP, PNN, SVM	Clinical variables	NR	Missing data handling/imbalance addressing/3fold cross-validation	PNN: 0.787 ± 0.27, RBF: 0.686, MLP: 0.638, SVM: 0.416 ± 0.27

*AIF* arterial input function, *ANN* artificial neural network, *AUC* area under the curve, *CBCT* cone-beam computed tomography, *CBF* cerebral blood flow, *CBV* cerebral blood volume, *CI* confidence interval, *CNN* convolutional neural network, *CP* cerebral perfusion, *CT* computed tomography, *DCNN* deep convolutional neural network, *DBSE* dual-branch separation and enhancement, *DECT* dual-energy computed tomography, *DMTC* DWI & MTT & TTP & Clinical, DMTC* DWI & MTT & TTP & clinical of ext. val. set, *DT* decision tree, *DWI* diffusion-weighted imaging, *GBDT* gradient boosted decision tree, *HU* Hounsfield units, *KNN* k-nearest neighbors, *LASSO* least absolute shrinkage and selection operator, *LR* logistic regression, *MCA* middle cerebral artery, *MRI* magnetic resonance imaging, *MT* MTT&TTP, *MTT* mean transit time, *MLRA* multivariate logistic regression analysis, *NCCT* non-contrast computed tomography, *NN* neural network, *NR* not reported, *PNN* probability neural network, *PWI* perfusion weighted imaging, *RBF* radial basis function, *RF* random forest, *ROI* region of interest, *SD* standard deviation, *SGD* stochastic gradient descent, *SR-DA* spectral regression w/ discriminant analysis, *SR-KDA* spectral regression w/ kernel discriminant analysis, *SVM* support vector machine, *VOI* volume of interest, *WSDL* weekly supervised deep learning, *XGB* extreme gradient boosting

**Fig. 3:**
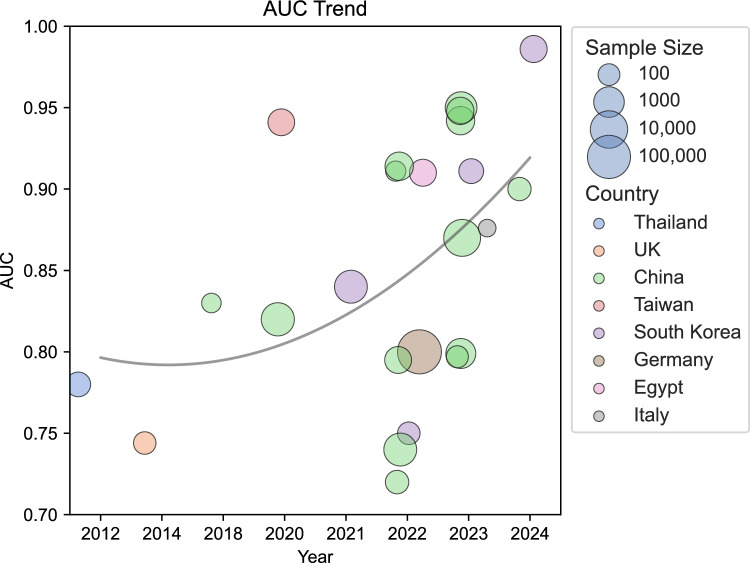
Characteristics of included studies

Figure 3 provides a detailed summary of the studies included in our review, highlighting key information such as sample sizes, countries of origin, publication years, best-reported AUC values, and the overall trends observed over time.

Table 3 summarizes the key findings, limitations, and recommendations for future research of the included studies in this systematic review.

**Table 3:** Machine learning studies in acute ischemic stroke: insights, limitations, and future directions for hemorrhagic transformation prediction

Study, (year)	Key findings	Limitations	Future suggestions
Heo et al. [23] (2024)	The Light Gradient Boosting model using all radiomics features outperformed the ExtraTrees model with textural features and the LR model with only clinical variables Radiomics feature extraction and model execution can be performed on consumer-grade CPUs, without requiring high computational power	Retrospective design and small sample size Differences in NCCT equipment, protocols, and parameters Lacks validation across different centers and regions Final patient groups were not perfectly matched due to exclusion of patients with image processing errors	Conduct multi-center validation studies Develop an ML model to automate VOI drawing, ensuring consistency and reducing manual variability Explore different ML models, such as XGBoost, to potentially enhance model performance Investigate the use of different clinical cutoffs to optimize model applicability in various clinical settings
Huang et al. [22] (2024)	The models can help stratify the risk of HT, allowing for individualized and accurate clinical treatment plans	Retrospective design and small sample size Focus on AIS patients with HT introduces selection bias and lowers statistical power	Validate findings with larger, multicenter, prospective studies Develop automatic segmentation techniques for cerebral hemorrhage Incorporate detailed clinical and hematological data and a wider range of AIS patients Investigate integrating models into clinical workflows to assess real-world impact Update models with new data and advanced techniques for better accuracy Use models to stratify patients for personalized treatment
Wen et al. [34] (2023)	No clinical or routine radiological factors were identified as predictors of HT, in contrast to some previous studies	Retrospective design and small sample size	Conduct multicenter, large-scale, and prospective studies to further validate and refine the predictive model
Ren et al. [24] (2023)	Superior accuracy of the combined clinical-radiomics model compared to models using only clinical data or radiomics Radiomics model based on NCCT image features improves HT risk prediction, linked to infarct size	Retrospective design and small sample K-nearest Neighbor for Incomplete Data: Methodological constraint Restricted Analysis of Radiomics Features	Explore radiomics-clinical outcomes link and expand clinical data in models Standardize CT imaging and incorporate post-thrombolysis MRI data Enhance the study of HT types and identify new risk factors Conduct multi-center studies for robust data and improve pt participation Establish collaborative networks and apply advanced AI for precise predictions
Ru et al. [16] (2023)	Subgroup Analysis of the WSDL Model showed superior performance in sICH cases (AUC: 0.833); lower in asymptomatic ICH and w/o ICH Useful for diagnosis and treatment in resource-limited settings Supports f/u and treatment planning, and aids decision-making	Risk of Overfitting: Due to a small number of positive cases Limited sICH cases HT detection flaws: HT is potentially underestimated by the NCCT method	Add aims to better assess HT Conduct Multicenter Studies: Increases model generalizability Use More Imaging Data: Includes CTP, SWI, and reperfusion as input variables for enhanced prediction accuracy
Heo et al. [25] (2023)	The developed model uses unprocessed raw DICOM images, simplifying the process DL w/ DECT shows potential for quick, automated prediction of HT post-EVT	Single-center study Included both symptomatic and asymptomatic hemorrhage cases Differences in detecting hemorrhage w/ CT vs. MRI Many pts were excluded due to DECT constraints Lacked a calculated statistical power for determining sample size	Intend to conduct a forward-looking, multi-institutional study to enhance the model's relevance Investigating extra elements besides DECT to better predict HT
Li et al. [26] (2023)	HT-Lab10 model (using XGB) showed high accuracy in Cohort 1 (AUC: 0.95) Effective in predicting both HT occurrence and in-hospital mortality post-HT (AUC: 0.85) Cohort 2 val. confirms the model's reliability for HT predictions and post-HT mortality assessment	Generalizability concerns due to the small, single-center sample in the Chinese population Inability to predict long-term mortality from incomplete f/u Reliance solely on lab data Technical limitations prevented the exploration of ensemble ML models	Test results in broader, varied groups Examine long-term death risk over extended periods Use medical imaging data to increase precision Explore combining various AI models for better outcomes
Da Ros et al. [36] (2023)	Deepens understanding of CB-CT images after EVT using ML Highlights standardized CB-CT analysis need for consistency and personalized care, despite early ML limitations	Small sample size Limited Imaging f/u: Used only 24-h post-procedure NCCT Higher symptomatic hemorrhage rate (11%); bias from excluding pts w/o CB-CT	Transition to DL for enhanced CB-CT prediction due to small sample sizes Develop robust DL tools for CB-CT analysis Standardize quantitative CB-CT analysis for improved precision in medicine
Jiang et al. [15] (2023)	DMTC model, combining DWI, MTT, TTP, and clinical data, accurately predicts HT in post-EVT AIS pts Clinical, DWI, and PWI data enhance AIS management via a DL model in EVT treatment VOI and slice data sets both effectively predict HT Slice data set, selected from axial MRI images based on VOI lesions, is easier to use, highly repeatable, and provides extensive image information The slice data set could potentially replace the VOI data set for DL model training	Small sample size, unclear optimal pts number for accuracy Data collected retro.ly, organized by admission time to resemble a pro. study Exclusion of MRI sequences T1WI, T2WI, FLAIR Included bridging therapy pts, despite similar HT rates across therapies	Boost model accuracy by enlarging sample size Use or emulate a pro. study design for stronger data Include additional MRI sequences for a thorough analysis Investigate how various therapy methods affect HT incidence and outcomes
Wen et al. [27] (2023)	MLP model suggested for post-thrombolysis hemorrhage risk prediction LR w/ lasso tops in AUC, MLP is second SVM and MLP are beneficial in Decision Curve Analysis Anticoagulation therapy is a negative predictor; rt-PA is positive	Sample limited to Northeast China, affecting generalizability Data issues could compromise individual pts outcome accuracy ML models used few variables	Increase sample size and diversify locations Improve data sharing and transparency Add more relevant variables to models Refine models for enhanced clinical usefulness
Bonkhoff et al. [12] (2022)	Early identification of high-risk pts improves personalized care Stroke severity is the most significant predictor GB models outperform LR	Limited data prevented DL use Basic clinical data and observational design Missing data (up to 7%)	Expand prediction scope to include various stroke-related complications Intend regular updates to the model; essential to validate w/ independent data sets
Elsaid et al. [35] (2022)	RFC and GBC models effectively forecast HT Main HT predictors: NIH stroke scale, infarction size, microbleeds	Generalizability is limited to specific hospitals and races Small sample; rt-PA therapy pts excluded	Conduct the study in various environments and include pts receiving rt-PA treatment Include a wider variety of biological and imaging indicators in the study
Xu et al. [28] (2022)	RF outperforms other models in predicting HT SHAP values improve clinical understanding of the RF model High Accuracy: The model predicts HT in post-alteplase stroke pts w/ 66.7% sensitivity and 80.7% specificity	Study limited to one center; broader val. needed Data set was too small for effective sICH analysis The algorithm might overlook key relationships Lack of SWI possibly undervalues HT risk	Conduct larger, multi-center pro. studies to confirm research results Improve algorithms by including a broader set of features, beyond usual physician assessments Implement SWI in future studies for precise HT estimation
Liu et al. [29] (2022)	Created a fast, efficient tool to screen pts at high risk for sICH Pinpointed major predictors of sICH in different areas The SVM model effectively predicted sICH in both Caucasian and Chinese pt groups	VISTA trials' Caucasian sample is not widely representative Han Chinese sample small and from one hospital Missing data	Develop clinical software Perform multi-center Chinese studies for val. And Smaller number of sICH in samples
Meng et al. [17] (2022)	Multi-parametric MRI models outperform single-sequence models Single-sequence accuracy order: ADC > TTP > CBF > CBV > MTT Model accuracy peaks w/ 14 features, then declines Overfitting occurs w/ too many features Combining MRI radiomics and clinical factors yields high accuracy (AUC 0.911)	Single-center study Complex image processing w/ basic radiomics Applicable only to non-lacunar acute cerebral infarcts	Validate models w/ multi-center datasets Use DL for better feature extraction Study cerebral atrophy's role in AIS and HT prediction Focus on early prediction of stroke complications
Cui et al. [30] (2022)	Created a practical CDSS prototype w/ Python Featuring a browser/server architecture for better integration and portability Suitable for web-based hospital systems	Image compression in comics can lead to loss of features Research is limited by population and data size	Utilize DL to improve predictions Broaden data and study groups for more universal applicability Apply ongoing learning for model updates Develop and evaluate the CDSS prototype consistently
Xie et al. [20] (2022)	The Rad-score, based on five radiomics features, was the sole predictor for HT Model accuracy for predicting HT varied w/ infarct sizes and treatment method	Small sample size and retro. Study design CT resolution constraints affected infarct area segmentation Excluded cerebral infarct volume on NCCT	Validate the model in a pros. multicenter setting Use larger sample sizes for robust val Focus on the effect of infarct size in massive cerebral stroke on HT Recognize limitations in hyperacute AIS pts Recommend cerebral perfusion imaging for deeper analysis
Wang et al. [21] (2022)	DBSE-Net for accurate lesion-HT prediction w/ dual-branch encoding Uses multi-scale features for NCCT-based HT prediction Algorithm addresses weak lesion features w/ key frame and adaptive encoding Efficiently extracts key information from NCCTs	NR	NR
Choi et al. [31] (2021)	ANN surpassed other ML algorithms w/ 0.844 accuracy in HT prediction post-AIS Scaling and resampling didn't improve ANN's HT prediction in AIS	Evaluated only clinic-demographic factors and initial lab variables Did not fully assess post-stroke management and HT radiologic markers	Incorporate post-stroke care factors in analysis Analyze radiologic indicators via DL in CT/MRI images Boost prediction accuracy w/ ensemble learning from clinical and imaging data
Chung et al. [32] (2020)	ANNs match expert-level stroke diagnosis They require little statistical training and uncover complex patterns Enhance prediction of sICH and mortality in AIS after rt-PA treatment Aid in personalized AIS treatment stratification	Retro. Design, small sample sized and single center study Potential underestimation of severity due to exclusion of pts on specific treatments	Conduct multi-center studies for wider applicability Implement pros. studies to develop evolving AI tools Prioritize accuracy in prediction tools and decision support systems
Wang et al. [33] (2020)	The best model is a three-layer neural network, achieving an AUC of 0.82 System deployment cut CT-to-treatment time from 52 to 41 min, significant at p < 0.001 Perfect identification of sICH cases as high-risk ML model reliably predicts personalized sICH risk post-stroke thrombolysis, enhancing treatment efficiency	Sample mainly from Northeast China, reducing generalizability Predictive accuracy is potentially limited due to data scope Models built using a narrow range of variables	Increase sample size and diversity for wider val Enhance data quality and transparency in upcoming studies Incorporate a broader range of variables in new models Address existing limitations and refine models
Yu et al. [10] (2018)	Extract HT imaging markers from PWI images w/o pre-established metrics Assessed using f/u GRE for insights into pre-EVT Kernel spectral regression shows highest accuracy (83.7 ± 2.6%)	Advanced preprocessing to address noise-related prediction errors Regularization to improve co-registration and label accuracy Multi-center evaluation for diverse recanalization cases Issue of small sample size Importance sampling or boosting over random sampling	Create a brain region atlas for HT likelihood assessment Merge atlas w/ eloquence features for better outcome prediction Use nonlinear models for AIS HT prediction complexity Add more physiological variables for model enhancement Quantitatively study factors affecting HT for reliable predictions
Bentley et al. [19] (2014)	SVM-based ML outperforms in predicting sICH ML more accurate than radiology in detecting thrombolysis-related sICH from CT scans ML via CT scans surpasses traditional methods in sICH prediction	A small percentage (5%) of sICH cases in a single-center study Difficulties in choosing features and risk of overfitting in small datasets Time-intensive and inefficient image processing procedures	Validate ML in thrombolysis w/ larger, diverse studies Acquire unbiased data pros.ly, including diverse pts and untreated potential cases Tackle challenges of imbalanced datasets; optimize image-space features w/caution Enhance image-processing efficiency for clinical applicability
Dharmasaroja et al. [18] (2012)	AUC analysis showed no significant difference between RBF, MLP, and PNN models, while PNN outperformed SVM Three models identified stroke subtype as an important predictor for sICH, along w/ other factors like stroke location, prothrombin time, etc Using multiple ANN models showed advantages over a single model	NR	NR

*ANN* artificial neural network, *ADC* apparent diffusion coefficient, *AUC* area under the curve, *AIS* acute ischemic stroke, *CBCT* cone-beam computed tomography, *CBF* cerebral blood flow, *CBV* cerebral blood volume, *CTP* computed tomography perfusion, *CT* computed tomography, *CDSS* clinical decision support system, *DECT* dual-energy computed tomography, *DL* deep learning, *DMTC* DWI & MTT & TTP & Clinical, *DWI* diffusion-weighted imaging, *EVT* endovascular treatment, *GB* gradient boosting, *HT* hemorrhagic transformation, *ICH* intracerebral hemorrhage, *IVT* IV thrombolysis, *LR* logistic regression, *ML* machine learning, *MLP* multilayer perceptron, *MRI* magnetic resonance imaging, *MT* MTT&TTP, *MTT* mean transit time, *NCCT* non-contrast computed tomography, *NIHSS* National Institutes of Health Stroke Scale, *NR* not reported, *PNN* probability neural network, *PWI* perfusion weighted MRI, *RBF* radial basis function, *RF* random forest, *sICH* symptomatic ICH, *SHAP* SHapley Additive exPlanations, *SVM* support vector machine, *SWI* susceptibility weighted imaging, *TT* thrombin time, *TTP* time to peak, *VOI* volume of interest, *WSDL* weekly supervised deep learning, *XGB* extreme GB

## Discussion

HT, a life-threatening complication after AIS, is an important contributor to morbidity and mortality [37]. Predicting the risk of HT at the time of admission can potentially assist healthcare providers in enhancing the quality of patient care. This predictive capability facilitates more informed decision-making. Currently, there are several well-established scoring systems designed to predict this complication. These systems primarily use clinical and imaging features to identify patients who are at increased risk [38–42]. However, these traditional scoring systems demonstrate only moderate performance in evaluating the risk of future HT [6, 43].

This systematic review evaluates the recently developed ML/DL models in forecasting HT after AIS. The findings from our investigation underscore the promising capability of ML and DL techniques in improving the risk estimation of HT following an AIS, surpassing conventional scoring systems in performance. Integrating clinical and imaging data can significantly improve the accuracy of HT prediction models. The model's effectiveness largely relies on the imaging methods used, with Meng et al. showing that multiparametric MRI techniques yield better predictions than single-sequence imaging methods [17].

The employment of radiomics features, which aims to extract quantitative and ideally reproducible information from diagnostic images, including intricate patterns not easily discernible or quantifiable by the human eye, holds significant importance as input factors for the development of ML algorithms in this field [44, 45]. As evidenced by various studies, the implementation of radiomics features on NCCT has shown promising performance. For instance, Heo et al. [23] demonstrated a notable improvement in model performance with a radiomics-based approach compared to models relying solely on clinical factors, with the radiomics model achieving nearly doubled performance (AUC of 0.986 vs. 0.544). Furthermore, Huang et al. [22] presented compelling results with two models: one integrating clinical and radiomics features, yielding a performance of 0.9, and another solely based on clinical features, achieving a lower performance of 0.6. These findings underscore the potential of incorporating radiomics features into ML frameworks to enhance diagnostic accuracy and prognostic capabilities in medical imaging analysis. Moreover, the observed superiority of radiomics features highlights the effectiveness of ensemble learning techniques in handling complex data patterns and optimizing model performance.

An interesting observation was that LR with the addition of LASSO (L1) regularization was able to beat other robust algorithms in some studies [12, 27]. This phenomenon signifies the fact that not always complex models outperform simpler ones. As to why LASSO regularization is so effective, it effectively shrinks the irrelevant coefficients to zero thus ‘selecting’ the most informative features [46].

In the study by Liu et al. [29], they tested their developed SVM model on two diverse populations: Caucasian and Chinese patients. They observed that the performance of the model in the Caucasian cohort was significantly higher than in the Chinese cohort. This finding highlights the necessity of adapting models to accommodate diverse genetic and environmental factors, thereby enhancing diagnostic accuracy across different ethnicities. The researchers attributed the performance disparity to factors such as small sample sizes and incomplete data, and they recommended conducting studies across multiple centers to confirm the results.

The comparative study of various ML models reveals distinct advantages and applications of each model in predicting HT risk. While simpler models like LR and DT are easier to understand, they typically underperform compared to more sophisticated methods like SVM and XGB.

Contrary to our expectations, the overall performance of DL-based methods was unexpectedly underwhelming, despite their complexity and recent successes. However, XGB and other ensemble DT-based models showcased better performance on tabular data, as demonstrated in an exploratory study by Grinsztajn et al. [47], a trend that was further supported by our review. One plausible explanation for this unexpected outcome could be the limited predictive cues visible in imaging studies of AIS patients, such as the hyperdense artery sign or ischemic changes [48]. However, models utilizing both clinical and imaging variables generally demonstrated improved performance [15, 17, 24]. In this theme, we observe that classic ML techniques fall short in terms of performance when fed high-dimensional data such as data derived from CT imaging [19], whereas CNN-based solutions shine in these scenarios [15]. As pointed out by Grinsztajn et al., ANNs are sensitive to irrelevant inputs, while tree-based solutions excel at these situations and are often used for this task (e.g., RF for feature selection) [47].

In the context of image processing, DL solutions especially CNN-based ones have dominated the field for the last 10 years [49]. It is possible to dodge using DL approaches by employing feature extraction methods such as radiomics, however, they tend to be less interpretable. The major advantage of CNNs over traditional hand-crafted methods is their ability to automatically learn relevant features directly from the data. However, this comes with its drawbacks. DL solutions, CNNs included, are data-hungry methods, requiring ample amounts of training data if one needs to train them from scratch [7]. Unfortunately, in the field of medicine, this is a scarcity. Thus, it is often seen those methods such as XGB and SVM shine the most with relatively limited data.

Over the years, several methods have been developed to increase the transparency of the ML/DL decision-making process. One of the most popular ones is Shapley values [50]. Xu et al. [28] utilized Shapley values to find the most influential factors for HT risk prediction. This method finds the most important input features using concepts from cooperative game theory. One of the most striking advantages of this method is its independence from the underlying predictive algorithm used, however, the caveat is the complexity of computing these values. Another method that is commonly used in vision models is GradCAM [51]. It uses the gradient of the classification score concerning some intermediate convolutional layers and effectively highlights the most salient regions in the input image. A significant barrier to the practical application of these solutions is their dependence on a large number of features, often ranging from dozens to hundreds, which can be impractical and expensive to supply.

The simplistic structure of medical data, such as binary indicators for diseases and basic age metrics, doesn't align well with the intricate input requirements of neural networks. This leads to a poor reflection of the actual biological diversity. Binary values for complex conditions like type 2 diabetes or hypertension reduce nuanced medical states to a mere ‘1’ or ‘0’, missing their broader biological effects. Similarly, linear age representation fails to mirror the non-linear nature of biological aging, overlooking the varied changes that occur at different life stages.

The success of DL, particularly in vision and language processing, is largely due to the vast availability of 'raw' data. Unlike traditional ML methods, DL operates in latent spaces, turning raw data into meaningful numerical forms. This shift became notable as neural networks grew more complex. In medical imaging, this success is due to the abundance of raw data available. For DL to be effectively used in healthcare, there needs to be a significant accumulation of diverse, unprocessed medical data, including images, physiological signals, and sensor data. Without such datasets, traditional ML techniques like RF and SVM will remain prevalent.

The accurate prediction of HT following AIS is paramount for guiding clinical decision-making and optimizing patient outcomes. In our study, GB demonstrated superior performance among all evaluated ML algorithms, achieving a median AUC of 0.91. This was followed by RF, LR, and SVM, which attained AUCs of 0.83, 0.77, and 0.76, respectively. Among the neural network models, CNNs had the best performance with a median AUC of 0.91, indicating comparable efficacy to GB. Furthermore, our evaluation revealed that in all studies where ML models were compared to traditional scoring systems, the ML models consistently exhibited superior performance. This suggests a significant advantage of utilizing advanced ML techniques over conventional methods.

The superior performance of certain ML and DL models, particularly those incorporating both clinical and imaging variables, underscores their potential utility as adjunctive tools for clinicians. Implementation of these advanced predictive models could facilitate early identification of patients at heightened risk of HT, enabling timely interventions and personalized treatment strategies to mitigate this critical complication and improve prognosis. Thus, this systematic review not only contributes to the scientific understanding of HT prediction models but also has tangible implications for improving patient care and outcomes in AIS management. Creating user-friendly Clinical Decision Support Systems (CDSS) and integrating ML/DL models into hospital systems could enhance the efficiency and reach of stroke care, particularly in less-equipped areas, thereby narrowing the gap in healthcare delivery. However, widespread clinical adoption faces challenges like ensuring the models' relevance for diverse populations, demystifying complex ML techniques, and avoiding overfitting through careful model selection and validation. Moreover, effectively integrating these technologies into daily medical practices is crucial, which involves simplifying data entry, continuously updating the models, and developing accessible interfaces for healthcare professionals.

In this systematic review, a major limitation was the significant diversity among the models studied and their input variables, which made a meta-analysis impractical. Nonetheless, despite this limitation, we were able to draw meaningful conclusions by comparing various models with each other and with conventional scoring systems and illustrating a clear visualization of the developed models.

The reviewed studies also had several limitations, including small sample size and retrospective design which potentially increase selection bias and affect the generalizability. Methodological issues, such as the use of specific algorithms like K-nearest Neighbor (KNN) for missing data and limited radiomics analysis, reduced the depth of the studies. Furthermore, the majority of studies were single-center studies with few HT cases introduced biases and limited the research scope. Technical and diversity limitations also hindered the models' predictive accuracy and applicability across various populations.

To enhance the precision and versatility of predictive models, future research should integrate larger, diverse sample sizes and adopt prospective, multicenter study designs to capture a wide range of clinical scenarios and patient demographics. Incorporating diverse ethnicities is essential to address population heterogeneity and reduce biases. Efforts should focus on integrating multimodal data while standardizing imaging protocols to ensure consistency and reproducibility. Enhancing model transparency through explainable AI (XAI) techniques will improve interpretability and trust. Rigorous validation using comprehensive performance metrics is also crucial. These strategies will significantly improve the performance, applicability, and reliability of ML and DL models in clinical practice.

## Supplementary Information

Below is the link to the electronic supplementary material.

Supplementary file1 (DOCX 72 kb)

## Data Availability

The data of the current study are available from the corresponding author on reasonable request.
