# Supplementary material for: Machine learning and deep learning algorithms in stroke medicine: a systematic review of hemorrhagic transformation prediction models
Source: J Neurol. 2024 Dec 12;272(1):37. doi: 10.1007/s00415-024-12810-6 (PMC11638292; doi:10.1007/s00415-024-12810-6)
Supplement: Supplementary file 1 — Supplementary file1 (DOCX 72 kb) [file 415_2024_12810_MOESM1_ESM.docx]

# **Supplementary Material**

**Machine Learning and Deep Learning Algorithms in Stroke Medicine: A Systematic Review of Hemorrhagic Transformation Prediction Models**

**Journal: Journal of Neurology**

- Mahbod Issaiy*, M.D., Email address: [mahbodissaiy@gmail.com](mailto:mahbodissaiy@gmail.com)

Affiliation: Advanced Diagnostic and Interventional Radiology Research Center (ADIR), Tehran University of Medical Sciences, Tehran, Iran

- Diana Zarei*, M.D., Email address: [dianazarei1997@gmail.com](mailto:dianazarei1997@gmail.com)

Affiliation: Advanced Diagnostic and Interventional Radiology Research Center (ADIR), Tehran University of Medical Sciences, Tehran, Iran

- Shahriar Kolahi, M.D., Email address: [shahriar.kolahi@gmail.com](mailto:shahriar.kolahi@gmail.com)

Affiliation: Advanced Diagnostic and Interventional Radiology Research Center (ADIR), Tehran University of Medical Sciences, Tehran, Iran

- David S Liebeskind, M.D., FAAN, FAHA, FANA, FSVIN, FWSO (Corresponding author), Email address: [davidliebeskind@yahoo.com](mailto:davidliebeskind@yahoo.com)

Affiliation: Department of Neurology, University of California, Los Angeles, CA, USA

Address: Neuroscience Research Building, 635 Charles E Young Drive South, Suite 225 Los Angeles, CA 90095-7334, Phone: 310-963-5539, ORCID ID: 0000-0002-5109-8736

*Mahbod Issaiy and Diana Zarei contributed equally as the first author.

*Table S1. PRISMA checklist*

| **Section and Topic** | **Item #** | **Checklist item** | **Location where item is reported** |
| --- | --- | --- | --- |
| **TITLE** | | |  |
| Title | 1 | Identify the report as a systematic review. | Page 1 |
| **ABSTRACT** | | |  |
| Abstract | 2 | See the PRISMA 2020 for Abstracts checklist. | Page 2 |
| **INTRODUCTION** | | |  |
| Rationale | 3 | Describe the rationale for the review in the context of existing knowledge. | Page 3 |
| Objectives | 4 | Provide an explicit statement of the objective(s) or question(s) the review addresses. | Pages 2, 3, 4 |
| **METHODS** | | |  |
| Eligibility criteria | 5 | Specify the inclusion and exclusion criteria for the review and how studies were grouped for the syntheses. | Page 5 |
| Information sources | 6 | Specify all databases, registers, websites, organisations, reference lists and other sources searched or consulted to identify studies. Specify the date when each source was last searched or consulted. | Page 4 |
| Search strategy | 7 | Present the full search strategies for all databases, registers and websites, including any filters and limits used. | Supplementary Material, Table S2 |
| Selection process | 8 | Specify the methods used to decide whether a study met the inclusion criteria of the review, including how many reviewers screened each record and each report retrieved, whether they worked independently, and if applicable, details of automation tools used in the process. | Pages 4, 5 |
| Data collection process | 9 | Specify the methods used to collect data from reports, including how many reviewers collected data from each report, whether they worked independently, any processes for obtaining or confirming data from study investigators, and if applicable, details of automation tools used in the process. | Pages 4, 5 |
| Data items | 10a | List and define all outcomes for which data were sought. Specify whether all results that were compatible with each outcome domain in each study were sought (e.g. for all measures, time points, analyses), and if not, the methods used to decide which results to collect. | Page 5 |
|  | 10b | List and define all other variables for which data were sought (e.g. participant and intervention characteristics, funding sources). Describe any assumptions made about any missing or unclear information. | Page 5 |
| Study risk of bias assessment | 11 | Specify the methods used to assess risk of bias in the included studies, including details of the tool(s) used, how many reviewers assessed each study and whether they worked independently, and if applicable, details of automation tools used in the process. | Page 5 |
| Effect measures | 12 | Specify for each outcome the effect measure(s) (e.g. risk ratio, mean difference) used in the synthesis or presentation of results. | NA |
| Synthesis methods | 13a | Describe the processes used to decide which studies were eligible for each synthesis (e.g. tabulating the study intervention characteristics and comparing against the planned groups for each synthesis (item #5)). | NA |
|  | 13b | Describe any methods required to prepare the data for presentation or synthesis, such as handling of missing summary statistics, or data conversions. | Page 5 |
|  | 13c | Describe any methods used to tabulate or visually display results of individual studies and syntheses. | Page 5 |
|  | 13d | Describe any methods used to synthesize results and provide a rationale for the choice(s). If meta-analysis was performed, describe the model(s), method(s) to identify the presence and extent of statistical heterogeneity, and software package(s) used. | Page 5 |
|  | 13e | Describe any methods used to explore possible causes of heterogeneity among study results (e.g. subgroup analysis, meta-regression). | NA |
|  | 13f | Describe any sensitivity analyses conducted to assess robustness of the synthesized results. | NA |
| Reporting bias assessment | 14 | Describe any methods used to assess risk of bias due to missing results in a synthesis (arising from reporting biases). | NA |
| Certainty assessment | 15 | Describe any methods used to assess certainty (or confidence) in the body of evidence for an outcome. | NA |
| **RESULTS** | | |  |
| Study selection | 16a | Describe the results of the search and selection process, from the number of records identified in the search to the number of studies included in the review, ideally using a flow diagram. | Page 6 |
|  | 16b | Cite studies that might appear to meet the inclusion criteria, but which were excluded, and explain why they were excluded. | Page 6 |
| Study characteristics | 17 | Cite each included study and present its characteristics. | Pages 7-10 |
| Risk of bias in studies | 18 | Present assessments of risk of bias for each included study. | Page 7 |
| Results of individual studies | 19 | For all outcomes, present, for each study: (a) summary statistics for each group (where appropriate) and (b) an effect estimate and its precision (e.g. confidence/credible interval), ideally using structured tables or plots. | Pages 6-25 |
| Results of syntheses | 20a | For each synthesis, briefly summarise the characteristics and risk of bias among contributing studies. | Pages 6-25 |
|  | 20b | Present results of all statistical syntheses conducted. If meta-analysis was done, present for each the summary estimate and its precision (e.g. confidence/credible interval) and measures of statistical heterogeneity. If comparing groups, describe the direction of the effect. | Pages 6-25 |
|  | 20c | Present results of all investigations of possible causes of heterogeneity among study results. | Pages 6-25 |
|  | 20d | Present results of all sensitivity analyses conducted to assess the robustness of the synthesized results. | Pages 6-25 |
| Reporting biases | 21 | Present assessments of risk of bias due to missing results (arising from reporting biases) for each synthesis assessed. | Pages 6-25 |
| Certainty of evidence | 22 | Present assessments of certainty (or confidence) in the body of evidence for each outcome assessed. | NA |
| **DISCUSSION** | | |  |
| Discussion | 23a | Provide a general interpretation of the results in the context of other evidence. | Pages 25-29 |
|  | 23b | Discuss any limitations of the evidence included in the review. | Pages 25-29 |
|  | 23c | Discuss any limitations of the review processes used. | Pages 25-29 |
|  | 23d | Discuss implications of the results for practice, policy, and future research. | Pages 25-29 |
| **OTHER INFORMATION** | | |  |
| Registration and protocol | 24a | Provide registration information for the review, including register name and registration number, or state that the review was not registered. | Page 4 |
|  | 24b | Indicate where the review protocol can be accessed, or state that a protocol was not prepared. | Page 4 |
|  | 24c | Describe and explain any amendments to information provided at registration or in the protocol. | Page 4 |
| c | 25 | Describe sources of financial or non-financial support for the review, and the role of the funders or sponsors in the review. | Page 30 |
| Competing interests | 26 | Declare any competing interests of review authors. | Page 30 |
| Availability of data, code and other materials | 27 | Report which of the following are publicly available and where they can be found: template data collection forms; data extracted from included studies; data used for all analyses; analytic code; any other materials used in the review. | Page 30 |

*Table S2. Search Strategy*

| **Database** | **Search Strategy** | **Results** |
| --- | --- | --- |
| **PubMed** | ("Brain Ischemia"[MeSH Terms] OR "ischemic stroke*" OR "cerebral infarct*" OR "cerebrovascular accident*" OR "cerebral ischemi*" OR "acute ischemic stroke*" OR "thrombotic stroke*" OR "embolic stroke*" OR "cerebrovascular ischemi*" OR "occlusive cerebrovascular diseas*") AND ("Artificial Intelligence"[MeSH Terms] OR "Machine Learning"[MeSH Terms] OR "neural network*" OR "deep learn*" OR "predictive model*" OR "computational learn*" OR "data min*" OR "supervised learn*" OR "unsupervised learn*" OR "reinforcement learn*" OR "algorithmic learn*" OR "feature extract*" OR "pattern recogni*" OR "convolutional neural network*" OR "deep neural network*" OR "machine intellig*" OR "decision tree learn*" OR "random forest*" OR "support vector machine*" OR "ensemble learn*") AND ("Cerebral Hemorrhage"[MeSH Terms] OR "hemorrhagic transformation*" OR "hemorrhagic conversion*" OR "intracerebral hemorrhage*" OR "secondary hemorrhage*") | 103 |
| **Embase** | ('brain ischemia' OR 'ischemic stroke' OR 'cerebral infarction' OR 'cerebral infarct*' OR 'cerebrovascular accident' OR 'cerebral ischemia' OR 'acute ischemic stroke' OR 'thrombotic stroke' OR 'embolic stroke' OR 'cerebrovascular ischemia' OR 'occlusive cerebrovascular disease') AND ('artificial intelligence' OR 'machine learning' OR 'neural network' OR 'deep learning' OR 'predictive modeling' OR 'supervised learning' OR 'unsupervised learning' OR 'reinforcement learning' OR 'algorithmic learning' OR 'feature extraction' OR 'pattern recognition' OR 'convolutional neural network' OR 'deep neural network' OR 'machine intelligence' OR 'decision tree' OR 'random forest' OR 'support vector machine' OR 'ensemble learning') AND ('cerebral hemorrhage' OR 'hemorrhagic transformation' OR 'hemorrhagic conversion' OR 'intracerebral hemorrhage' OR 'secondary hemorrhage') | 1052 |
| **Web of Science (ISI)** | TS=("ischemic stroke" OR "cerebral infarction" OR "cerebrovascular accident" OR "cerebral ischemia" OR "acute ischemic stroke" OR "thrombotic stroke" OR "embolic stroke" OR "cerebrovascular ischemia" OR "occlusive cerebrovascular disease") AND TS=("artificial intelligence" OR "machine learning" OR "neural networks" OR "deep learning" OR "predictive modeling" OR "computational learning" OR "data mining" OR "supervised learning" OR "unsupervised learning" OR "reinforcement learning" OR "algorithmic learning" OR "feature extraction" OR "pattern recognition" OR "convolutional neural networks" OR "deep neural networks" OR "machine intelligence" OR "decision tree learning" OR "random forests" OR "support vector machines" OR "ensemble learning") AND TS=("hemorrhagic transformation" OR "hemorrhagic conversion" OR "intracerebral hemorrhage" OR "secondary hemorrhage") | 70 |
| **Scopus** | TITLE-ABS-KEY ("ischemic stroke" OR "cerebral infarction" OR "cerebrovascular accident" OR "cerebral ischemia" OR "acute ischemic stroke" OR "thrombotic stroke" OR "embolic stroke" OR "cerebrovascular ischemia" OR "occlusive cerebrovascular") AND TITLE-ABS-KEY ("artificial intelligence" OR "machine learning" OR "neural network" OR "deep learning" OR "predictive modeling" OR "supervised learning" OR "unsupervised learning" OR "reinforcement learning" OR "algorithmic learning" OR "feature extraction" OR "pattern recognition" OR "convolutional neural network" OR "deep neural network" OR "machine intelligence" OR "decision tree" OR "random forest" OR "support vector machine" OR "ensemble learning") AND TITLE-ABS-KEY ("cerebral hemorrhage" OR "hemorrhagic transformation" OR "hemorrhagic conversion" OR "intracerebral hemorrhage" OR "secondary hemorrhage") | 160 |
| **IEEE** | ("ischemic stroke" OR "cerebral infarction" OR "cerebrovascular accident" OR "cerebral ischemia" OR "acute ischemic stroke" OR "thrombotic stroke" OR "embolic stroke" OR "cerebrovascular ischemia" OR "occlusive cerebrovascular") AND ("artificial intelligence" OR "machine learning" OR "neural network" OR "deep learning" OR "predictive modeling" OR "supervised learning" OR "unsupervised learning" OR "reinforcement learning" OR "algorithmic learning" OR "feature extraction" OR "pattern recognition" OR "convolutional neural network" OR "deep neural network" OR "machine intelligence" OR "decision tree" OR "random forest" OR "support vector machine" OR "ensemble learning") AND ("cerebral hemorrhage" OR "hemorrhagic transformation" OR "hemorrhagic conversion" OR "intracerebral hemorrhage" OR "secondary hemorrhage") | 29 |

, onset to door time; PWI, perfusion-weighted imaging; Pros, prospective; Retro, retrospective; SD, standard deviation; sICH, symptomatic ICH; SAH, sub-arachnoid hemorrhage; SWI, susceptibility weighted imaging

*Table S3. Quality Assessment (PROSPERO)*

| **Study** | **Classify the type of prediction model evaluation** | **DOMAIN-1: Participants A. Risk of Bias** | **DOMAIN-1: Participants B. Applicability Concern** | **DOMAIN-2: Predictors A. Risk of Bias** | **DOMAIN-2: Predictors B. Applicability Concern** | **DOMAIN-3: Outcome A. Risk of Bias** | **DOMAIN-3: Outcome B. Applicability Concern** | **DOMAIN-4: Analysis Risk of Bias** | **Step-4: Overall Risk of Bias** | **Step-4: Overall Applicability Concern** |
| --- | --- | --- | --- | --- | --- | --- | --- | --- | --- | --- |
| Heo et al. [1](2024) | Internal validation | Low | Low | Low | Low | Low | Low | Low | Low | Low |
| Huang et al. [2](2024) | Internal validation | Low | Low | Low | Low | Low | Low | Low | Low | Low |
| Wen et al. [3] (2023) | Internal Validation | Low | Low | Low | Low | Low | Low | Low | Low | Low |
| Ren et al. [4](2023) | Internal and External Validation | Low | Low | Low | Low | Low | Low | Low | Low | Low |
| Jiang et al. [5] (2023) | Internal and External Validation | **High** | Low | Low | Low | Low | Low | **High** | **High** | Low |
| Ru et al. [6](2023) | Internal Validation | **High** | Low | Low | Low | Low | Low | **High** | **High** | Low |
| Da Ros et al. [7](2023) | Internal Validation | Low | Low | Low | Low | Low | Low | Low | Low | Low |
| Li et al. [8](2023) | Internal and External Validation | Low | Low | Low | Low | Low | Low | Low | Low | Low |
| Wen et al. [9](2023) | Internal and External Validation | Low | Low | Low | Low | Low | Low | Low | Low | Low |
| Heo et al. [10] (2023) | Internal and External Validation | Low | Low | Low | Low | Low | Low | Low | Low | Low |
| Bonkhoff et al. [11](2022) | Internal Validation | Low | Low | Low | Low | Low | Low | Low | Low | Low |
| Elsaid et al. [12] (2022) | Internal Validation | Low | Low | Low | Low | Low | Low | Low | Low | Low |
| Xu et al. [13] (2022) | Internal Validation | Low | Low | Low | Low | Low | Low | Low | Low | Low |
| Liu et al. [14] (2022) | Internal and External Validation | Low | Low | Low | Low | Low | Low | Low | Low | Low |
| Meng et al. [15](2022) | Internal Validation | Low | Low | Low | Low | Low | Low | **High** | **High** | Low |
| Cui et al. [16](2022) | Internal and External Validation | Low | Low | Low | Low | Low | Low | Low | Low | Low |
| Xie et al. [17](2022) | Internal and External Validation | **High** | Low | Low | Low | Low | Low | **High** | **High** | Low |
| Wang et al. [18](2022) | Internal Validation | **High** | Unclear | Low | Low | Low | Unclear | Unclear | **High** | Unclear |
| Choi et al. [19](2021) | Internal Validation | Low | Low | Low | Low | Low | Low | Low | Low | Low |
| Chung et al. [20](2020) | Internal Validation | Low | Low | Low | Low | Low | Low | Low | Low | Low |
| Wang et al. [21](2020) | Internal and External Validation | Low | Low | Low | Low | Low | Low | Low | Low | Low |
| Yu et al. [22] (2018) | Internal Validation | Low | Low | Low | Low | Low | Low | Low | Low | Low |
| Bentley et al. [23](2014) | Internal Validation | **High** | **High** | Low | Low | Low | Low | **High** | **High** | Low |
| Dharmasaroja et al. [24](2012) | Internal Validation | Low | Low | Low | Low | Low | Low | **High** | **High** | Low |

***Reference***

1. Heo, J., Sim, Y., Kim, B.M., Kim, D.J., Kim, Y.D., Nam, H.S., Choi, Y.S., Lee, S.K., Kim, E.Y., and Sohn, B. (2024). Radiomics using non-contrast CT to predict hemorrhagic transformation risk in stroke patients undergoing revascularization. Eur Radiol. 10.1007/s00330-024-10618-6.

2. Huang, Y.H., Chen, Z.J., Chen, Y.F., Cai, C., Lin, Y.Y., Lin, Z.Q., Chen, C.N., Yang, M.L., Li, Y.Z., and Wang, Y. (2024). The value of CT-based radiomics in predicting hemorrhagic transformation in acute ischemic stroke patients without recanalization therapy. Front Neurol *15*, 1255621. 10.3389/fneur.2024.1255621.

3. Wen, X., Xiao, Y., Hu, X., Chen, J., and Song, F. (2023). Prediction of hemorrhagic transformation via pre-treatment CT radiomics in acute ischemic stroke patients receiving endovascular therapy. Br J Radiol *96*, 20220439. 10.1259/bjr.20220439.

4. Ren, H., Song, H., Wang, J., Xiong, H., Long, B., Gong, M., Liu, J., He, Z., Liu, L., Jiang, X., et al. (2023). A clinical-radiomics model based on noncontrast computed tomography to predict hemorrhagic transformation after stroke by machine learning: a multicenter study. Insights Imaging *14*, 52. 10.1186/s13244-023-01399-5.

5. Jiang, L., Zhou, L., Yong, W., Cui, J., Geng, W., Chen, H., Zou, J., Chen, Y., Yin, X., and Chen, Y.C. (2023). A deep learning-based model for prediction of hemorrhagic transformation after stroke. Brain Pathol *33*, e13023. 10.1111/bpa.13023.

6. Ru, X., Zhao, S., Chen, W., Wu, J., Yu, R., Wang, D., Dong, M., Wu, Q., Peng, D., and Song, Y. (2023). A weakly supervised deep learning model integrating noncontrasted computed tomography images and clinical factors facilitates haemorrhagic transformation prediction after intravenous thrombolysis in acute ischaemic stroke patients. Biomed Eng Online *22*, 129. 10.1186/s12938-023-01193-w.

7. Da Ros, V., Duggento, A., Cavallo, A.U., Bellini, L., Pitocchi, F., Toschi, N., Mascolo, A.P., Sallustio, F., Di Giuliano, F., Diomedi, M., et al. (2023). Can machine learning of post-procedural cone-beam CT images in acute ischemic stroke improve the detection of 24-h hemorrhagic transformation? A preliminary study. Neuroradiology *65*, 599-608. 10.1007/s00234-022-03070-0.

8. Li, X., Xu, C., Shang, C., Wang, Y., Xu, J., and Zhou, Q. (2023). Machine learning predicts the risk of hemorrhagic transformation of acute cerebral infarction and in-hospital death. Comput Methods Programs Biomed *237*, 107582. 10.1016/j.cmpb.2023.107582.

9. Wen, R., Wang, M., Bian, W., Zhu, H., Xiao, Y., He, Q., Wang, Y., Liu, X., Shi, Y., Hong, Z., and Xu, B. (2023). Machine learning-based prediction of symptomatic intracerebral hemorrhage after intravenous thrombolysis for stroke: a large multicenter study. Front Neurol *14*, 1247492. 10.3389/fneur.2023.1247492.

10. Heo, J., Yoon, Y., Han, H.J., Kim, J.J., Park, K.Y., Kim, B.M., Kim, D.J., Kim, Y.D., Nam, H.S., Lee, S.K., and Sohn, B. (2023). Prediction of cerebral hemorrhagic transformation after thrombectomy using a deep learning of dual-energy CT. Eur Radiol. 10.1007/s00330-023-10432-6.

11. Bonkhoff, A.K., Rübsamen, N., Grefkes, C., Rost, N.S., Berger, K., and Karch, A. (2022). Development and Validation of Prediction Models for Severe Complications After Acute Ischemic Stroke: A Study Based on the Stroke Registry of Northwestern Germany. J Am Heart Assoc *11*, e023175. 10.1161/jaha.121.023175.

12. Elsaid, A.F., Fahmi, R.M., Shehta, N., and Ramadan, B.M. (2022). Machine learning approach for hemorrhagic transformation prediction: Capturing predictors' interaction. Front Neurol *13*, 951401. 10.3389/fneur.2022.951401.

13. Xu, Y., Li, X., Wu, D., Zhang, Z., and Jiang, A. (2022). Machine Learning-Based Model for Prediction of Hemorrhage Transformation in Acute Ischemic Stroke After Alteplase. Frontiers in Neurology *13*, 897903. 10.3389/fneur.2022.897903.

14. Liu, J., Chen, X., Guo, X., Xu, R., Wang, Y., and Liu, M. (2022). Machine learning prediction of symptomatic intracerebral hemorrhage after stroke thrombolysis: a cross-cultural validation in Caucasian and Han Chinese cohort. Ther Adv Neurol Disord *15*, 17562864221129380. 10.1177/17562864221129380.

15. Meng, Y., Wang, H., Wu, C., Liu, X., Qu, L., and Shi, Y. (2022). Prediction Model of Hemorrhage Transformation in Patient with Acute Ischemic Stroke Based on Multiparametric MRI Radiomics and Machine Learning. Brain Sci *12*. 10.3390/brainsci12070858.

16. Cui, S., Song, H., Ren, H., Wang, X., Xie, Z., Wen, H., and Li, Y. (2022). Prediction of Hemorrhagic Complication after Thrombolytic Therapy Based on Multimodal Data from Multiple Centers: An Approach to Machine Learning and System Implementation. Journal of Personalized Medicine *12*, 2052.

17. Xie, G., Li, T., Ren, Y., Wang, D., Tang, W., Li, J., and Li, K. (2022). Radiomics-based infarct features on CT predict hemorrhagic transformation in patients with acute ischemic stroke. Front Neurosci *16*, 1002717. 10.3389/fnins.2022.1002717.

18. Wang, Z., Liu, Z., and Li, S. (2022). Weak lesion feature extraction by dual-branch separation and enhancement network for safe hemorrhagic transformation prediction. Comput Med Imaging Graph *97*, 102038. 10.1016/j.compmedimag.2022.102038.

19. Choi, J.M., Seo, S.Y., Kim, P.J., Kim, Y.S., Lee, S.H., Sohn, J.H., Kim, D.K., Lee, J.J., and Kim, C. (2021). Prediction of hemorrhagic transformation after ischemic stroke using machine learning. Journal of Personalized Medicine *11*. 10.3390/jpm11090863.

20. Chung, C.-C., Chan, L., Bamodu, O.A., Hong, C.-T., and Chiu, H.-W. (2020). Artificial neural network based prediction of postthrombolysis intracerebral hemorrhage and death. Scientific Reports *10*, 20501. 10.1038/s41598-020-77546-5.

21. Wang, F., Huang, Y., Xia, Y., Zhang, W., Fang, K., Zhou, X., Yu, X., Cheng, X., Li, G., Wang, X., et al. (2020). Personalized risk prediction of symptomatic intracerebral hemorrhage after stroke thrombolysis using a machine-learning model. Ther Adv Neurol Disord *13*, 1756286420902358. 10.1177/1756286420902358.

22. Yu, Y., Guo, D., Lou, M., Liebeskind, D., and Scalzo, F. (2018). Prediction of Hemorrhagic Transformation Severity in Acute Stroke From Source Perfusion MRI. IEEE Trans Biomed Eng *65*, 2058-2065. 10.1109/tbme.2017.2783241.

23. Bentley, P., Ganesalingam, J., Carlton Jones, A.L., Mahady, K., Epton, S., Rinne, P., Sharma, P., Halse, O., Mehta, A., and Rueckert, D. (2014). Prediction of stroke thrombolysis outcome using CT brain machine learning. Neuroimage Clin *4*, 635-640. 10.1016/j.nicl.2014.02.003.

24. Dharmasaroja, P., and Dharmasaroja, P.A. (2012). Prediction of intracerebral hemorrhage following thrombolytic therapy for acute ischemic stroke using multiple artificial neural networks. Neurol Res *34*, 120-128. 10.1179/1743132811y.0000000067.
